# The physiological basis and measurement of heart rate variability in humans

**DOI:** 10.1186/s40101-016-0113-7

**Published:** 2016-09-28

**Authors:** Adina E. Draghici, J. Andrew Taylor

**Affiliations:** 1 Department of Bioengineering, Northeastern University, Boston, MA USA; 2 Department of Physical Medicine and Rehabilitation, Harvard Medical School, Boston, MA USA; 3 Cardiovascular Research Laboratory, Spaulding Rehabilitation Hospital, 1575 Cambridge St, Cambridge, 02138 MA USA

**Keywords:** Heart rate variability, Respiratory sinus arrhythmia, Mayer waves, Autonomic control, Cardiovascular oscillations, Cardiac chronotropy, Frequency domain, Time domain

## Abstract

Cardiovascular variabilities were recognized over 250 years ago, but only in the past 20 years has their apparent utility come to be appreciated. Technological advancement has allowed precise measurement and quantification of short-term cardiovascular fluctuations; however, our understanding of the integrated mechanisms which underlie these oscillations is inadequate for their widespread application. Both autonomic branches, the parasympathetic and sympathetic nervous system, are key determinants of the magnitude of these spontaneous cardiovascular fluctuations. Heart rate variability can be an indicator of an individual cardiovascular condition. In this review, we will discuss the two primary rhythmic oscillations that underlie the complexity of the heart rate waveform. The first oscillation occurs over several cardiac cycles, is respiratory related, and termed respiratory sinus arrhythmia. The second oscillation occurs at an approximate 10 s cycle. Due to the closed-loop nature of the control system of cardiovascular oscillations, it is difficult to define specific relations among cardiovascular variables. In this review, we will present the feedforward and feedback mechanism that underlie both oscillations and their implication as quantitative measures of autonomic circulatory control. We will also review the various methodologies to assess them.

## Background

Variability in beat-by-beat heart period is an intrinsic characteristic of healthy cardiac functioning. This variability can reflect purposely generated responses to internal and external stimuli and may not reflect simple random fluctuations. For example, various stressors result in characteristic changes in variability and various disease states are associated with lesser variability. The primary regulators of cardiac chronotropy, the parasympathetic and sympathetic nervous systems, are key determinants of the magnitude of spontaneous cardiovascular variability. In this review, we will focus on two primary rhythmic oscillations occurring at the respiratory frequency and at the ~0.10 Hz frequency since these two have the greatest importance for human cardiovascular control. Moreover, these cardiovascular variabilities have been most studied and most often used to index autonomic circulatory control.

### Physiological basis

**Fig. 1:**
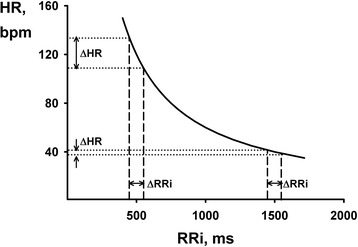
Relationship between heart rate (HR) and R-R interval (RRi). Note the hyperbolic behavior of HR (bpm) as a function of RRi (ms); same increase in RRi (ΔRRi = 100 ms) results in markedly different changes in HR (ΔHR) according to the chronotropic RRi

Cardiac chronotropy can be represented in two ways. Representation of cardiac chronotropy by heart rate (HR) as beats per minute (bpm) has a long history because it is readily and easily accessible by simple palpation of an artery. However, HR provides an estimate that is normalized to time (i.e., 60 s). With the development of the electrocardiogram (ECG), physiologists were able to minutely assess the time interval between beats in milliseconds. In fact, this linear measure of cardiac chronotropy better reflects its autonomic regulators. The interval between R waves in the ECG (RRi) is most commonly used and reflects a linear relationship to both parasympathetic (vagal) and sympathetic stimulation [1, 2]. Given that HR is the inverse of RRi, fluctuations in the two do not always conform to one another (Fig. 1). Hence, heart rate variability should not be used as anything more than a misnomer and instead RRi variability should be used. In this review, out of convenience and convention, we will use the widely adopted term heart rate variability (HRV) to discuss the physiology and measurement of RRi variability.

Fluctuations in heart rate are usually due to waxing and waning activity level in at least one arm of the autonomic nervous system (ANS). Under normal conditions, the chronotropic state of the heart is entirely regulated by the sinoatrial (SA) node. The SA node is directly innervated by both parasympathetic (vagal) and sympathetic efferents. Although both vagal and sympathetic nerves exert opposite chronotropic action on the heart, these effects are not symmetrical [2–4]. Vagal effects have a distinctly shorter latency than sympathetic effects. Vagally mediated RRi lengthening is mediated by synaptic release of acetylcholine (ACh). The response is almost immediate due to the very short effect latency and high turnover rate of ACh, allowing the parasympathetic system to exert cardiac control on a beat-by-beat basis [5]. Sympathetically mediated RRi shortening is mediated by synaptic release of noradrenaline which is reabsorbed and metabolized relatively slowly [5]. This results in a delay between the onset of sympathetic stimulation and the resultant changes in cardiac control. Hence, the effect of sympathetic nerves on RRi encompasses longer delays and potentially longer effects than parasympathetic nerves.

The characteristic delay and duration of the two autonomic arms result in different potential effects on HRV. That is, the faster vagal effects have the potential to act across a wide range of frequencies, from a single beat to many beats, whereas the sympathetic system may not. However, time domain quantification of HRV cannot discriminate frequency-specific effects. These can only provide global indexes for HRV. Frequency domain analysis can assess HRV across frequency components while ignoring random, non-rhythmic noise. While time domain is simple and global, the frequency domain eliminates noise and allows for easy differentiation of the two primary rhythmic oscillations contained in HRV. Hence, spectral analysis of HRV is the most precise and most widely used technique to assess specific rhythms that exist in the HRV. The majority of the data discussed below derives from frequency domain information.

### Respiratory frequency oscillations

Respiratory sinus arrhythmia (RSA) is a rhythmic heart rate oscillation at the respiratory frequency and can be quantitatively measured as a high-frequency component (usually >0.15 Hz) in the power spectrum of RRi. RSA is a widely studied physiological phenomenon that reflects numerous cardiorespiratory interactions. RSA is typically characterized by RRi shortening with inspiration and lengthening with expiration. The magnitude of RSA is thought to reflect the degree of respiratory modulation of vagal outflow and arises from complex interactions of both central and peripheral factors.

One possible driving mechanism for RSA is the response of RRi to arterial pressure fluctuations. During breathing, changes in intrathoracic pressure rhythmically alter venous return to the heart, thereby impacting cardiac output and subsequently changing arterial blood pressure. These arterial pressure changes engage the arterial baroreflex, generating oscillations in afferent activity to appropriately increase and decrease cardiac autonomic outflow, generating RSA. Indeed, in humans, respiration frequency and depth can strongly determine fluctuation amplitude [6–8]. The magnitude of RSA increases with increased tidal volume and decreased breathing frequency. It has been suggested that the magnitude of RSA might be due to respiratory changes in systolic blood pressure due to tidal volume and breathing frequency. Hence, it has been hypothesized that RSA represents baroreflex buffering of arterial blood pressure fluctuations induced by the mechanical effects of breathing.

**Fig. 2:**
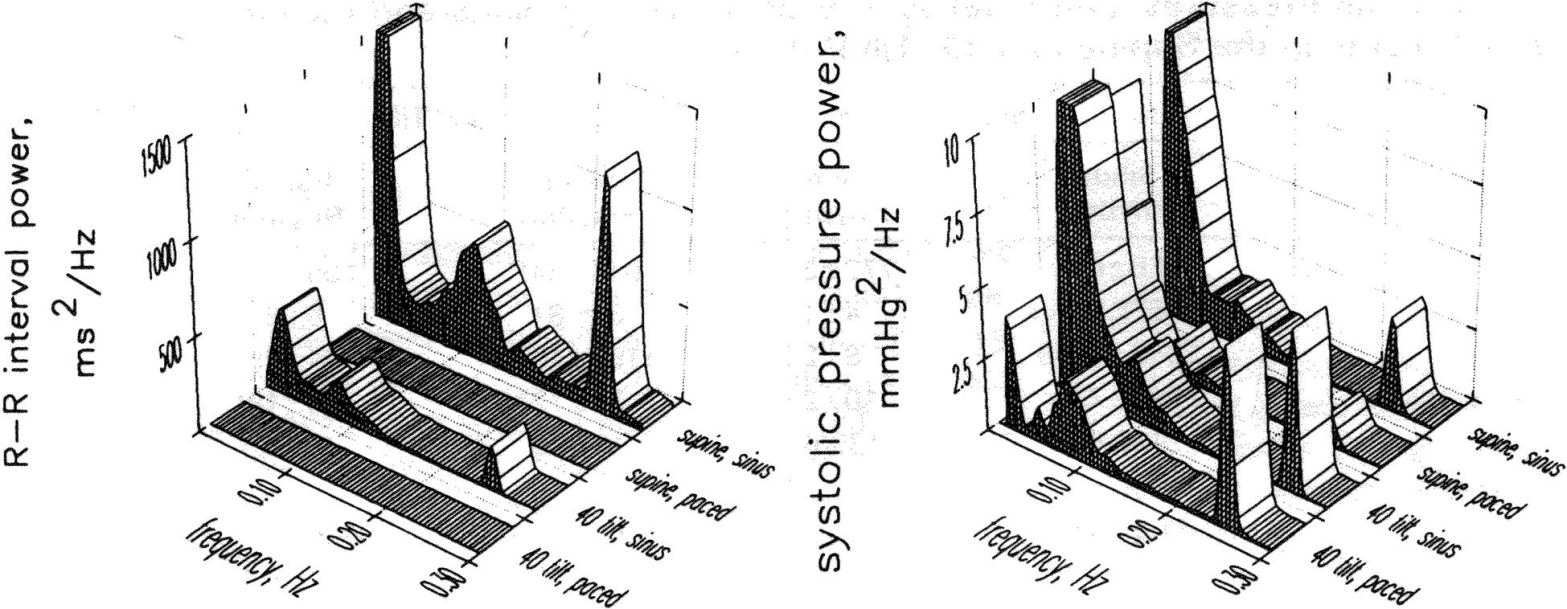
R-R interval and systolic pressure power in young individuals in the supine position and 40° tilt position with and without atrial pacing; elimination of RSA through fixed atrial pacing decreases pressure oscillations at the respiratory frequency in the supine position, but increases them in the upright position (redrawn from) [10]

If that were the case, eliminating RRi fluctuations, and hence RSA, should result in increased respiratory related arterial pressure fluctuations. However, in young individuals, elimination of RSA via complete autonomic blockade *decreased* arterial pressure fluctuations in the supine position, and it *increased* them in only the upright position [9]. These findings are further supported by elimination of RSA through fixed-rate atrial pacing. Atrial pacing decreased respiratory-related arterial pressure oscillations in the supine position, but increased them in the upright position (Fig. 2) [10]. In addition, it is apparent that RSA does not subserve a role in stabilizing diastolic pressure in supine humans. Progressive increases and decreases of RSA via low doses of the parasympatholytic agent atropine results in parallel and proportional increases and decreases in diastolic pressure oscillations at the respiratory frequency [11]. Hence, it seems that RSA contributes to arterial pressure oscillations in supine humans. Moreover, the postural differences can be explained by the shift in the phase relationship between RRi and systolic pressure oscillations. In the supine position, RRi oscillations are in phase with those in arterial pressure, but in the upright position, RRi oscillations follow those in arterial pressure [12]. This suggests that the arterial baroreflex is one important mechanism underlying RSA in upright but not supine humans. This could be due to the large caudal shift in blood volume consequent to assuming the upright posture that requires active and persistent baroreflex engagement to control arterial pressure [13].

However, baroreflex engagement does not explain the presence of RSA in supine humans. It is known that gating of excitatory input to the vagal cardiomotor neurons (CVM) partially contributes to RSA [14–16]. Data from animal models show CVM activity linked to the central respiratory cycle. While CVM activity in rats is greatest during inspiration [17], in cats, dogs, and humans, CVM are inhibited during inspiration and are mildly activated during expiration by stimulation of the arterial chemoreceptors and baroreceptors [18–20]. Thus, the susceptibility of CVM varies systematically during the respiratory cycle, and it is possible that RSA could arise from an efferent vagal oscillation that contributes to arterial pressure fluctuations by gating the excitatory input to the vagal motor neurons [3, 21]. The neural networks generating respiratory activity could not only drive respiratory motor neurons, but also impose patterns of activity in the vagal and sympathetic outflows. Functionally, this may provide a mechanism for the integration of cardiovascular and respiratory control [20].

Respiratory sinus arrhythmia is frequently used as an index of cardiac vagal tone or is even believed to be a direct measure of vagal tone. Substantial published data suggests that RSA quantifies tonic cardiac vagal activity [2, 22–26]. Despite physiological studies questioning respiratory sinus arrhythmia as a valid and reliable cardiac parasympathetic index [4, 27], numerous clinicians and experimentalists routinely estimate vagus nerve traffic to the heart with measurements of RSA. However, even though vagal outflow is the key contributor to HRV, there are significant caveats regarding the assumption that RSA is mediated exclusively by vagal mechanisms.

Observations that β-blockers augment RSA have been interpreted as indicating a central vagomimetic effect for these drugs [28, 29]. However, the data may actually suggest that sympathetic activity opposes vagally mediated RRi oscillations. This would mean that differences in RSA could not solely be ascribed to differences in vagal outflow. For example, β-adrenergic blocking drugs that do and do not cross the blood-brain barrier equally result in increased RSA [30]. Moreover, enhancement of RRi oscillations via cardioselective β-adrenergic blockade is exerted across a wide range of frequencies, from very low to high breathing frequencies [31]. If the delay and duration of sympathetic nervous effects constrain their impact to lower frequencies (<0.15 Hz), then cardiac sympathetic blockade should not impact variability across all frequencies. Moreover, in young healthy individuals, as vagal tone decreases during mild exercise, significant RSA can be generated by non-neural factors [32]. In dogs, direct cardiac vagus nerve stimulation that increases HRV does not result in a quantitative relationship between the frequency of vagal stimulation and HRV [33]. These results suggest that changes in HRV may not linearly reflect the vagally mediated chronotropic response and challenge the assumption that RSA is always a purely vagal mechanism.

### Low-frequency oscillations

Heart rate variability at frequencies slower than respiration in humans appear to occur in synchrony with arterial pressure Mayer waves, at frequencies as low as 0.03 Hz and up to 0.15 Hz, but generally close to 0.10 Hz, corresponding to a 10 s rhythm. Mayer waves result from an oscillation of sympathetic vasomotor tone [34–36]. Their amplitude has been presumed to measure vascular sympathetic activity since they are most apparent in response to sympathoexcitatory stimuli and are strongly attenuated or even abolished after acute α-adrenoceptor blockade [37–40]. However, these initial observations are not sufficient to capture the complex interactions that underlay the mechanism of Mayer wave oscillations.

Evidence suggests that the arterial baroreflex may be an essential component for sympathetic oscillations [12, 35, 36] and that Mayer waves are dependent upon intact arterial baroreflex function. The arterial baroreceptor reflex is the fastest and the most powerful regulator of blood pressure [41]. Thus, it has been proposed that Mayer wave oscillations could be caused by a time delay in the sympathetic feedback loop of the baroreflex [12, 42].

**Fig. 3:**
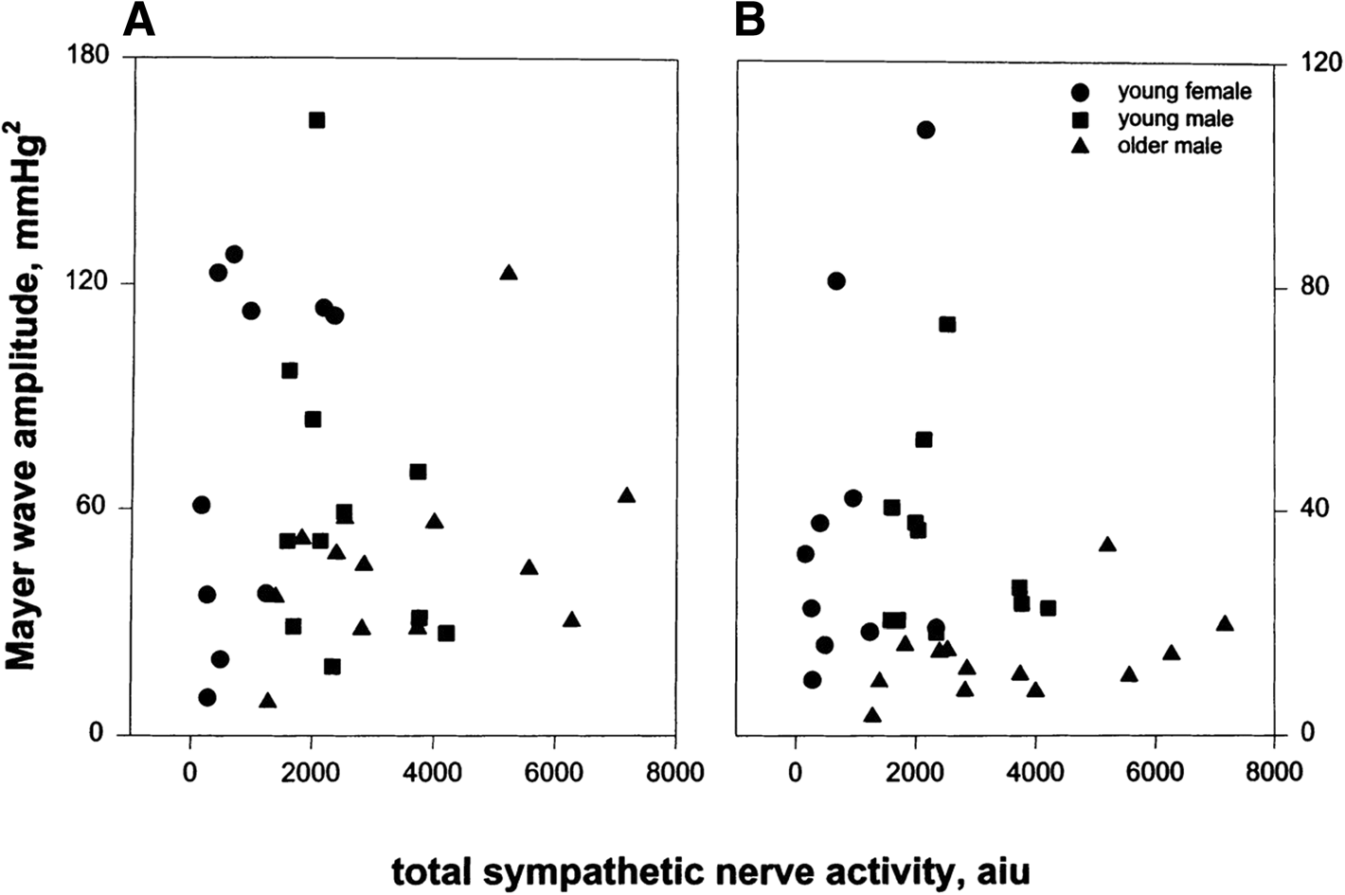
Relationship between vascular sympathetic outflow and Mayer wave amplitude in systolic (**a**) and diastolic pressure (**b**); despite striking differences in resting sympathetic outflow, older healthy males and young females show similar Mayer wave amplitudes (redrawn from [44]). *aiu* arbitrary integration units

In healthy individuals, monotonic atrial pacing in supine eliminates RRi variability, but does not affect low frequency arterial pressure oscillations (Fig. 2) [10]. This shows that elimination of RRi variability does not increase low-frequency arterial pressure oscillations. However, when vascular sympathetic outflow is increased by 40° tilt, elimination of low-frequency RRi variability resulted in greater diastolic pressure Mayer waves [10]. Thus, low-frequency RRi oscillations appear to buffer low-frequency arterial pressure oscillations only in upright humans. Hence, this might mean that a sympathetic mechanism is responsible, perhaps a latency in baroreflex-induced changes in vascular sympathetic outflow, in other words, a pressure-pressure feedback loop [43]. Thus, this supports the hypothesis that there is an arterial baroreflex link between Mayer wave oscillations in pressure and cardiac interval when sympathetic outflow is augmented. However, there is no simple linear relationship between Mayer wave amplitude and mean sympathetic outflow. Healthy individuals with different sympathetic outflow show no difference in Mayer wave amplitude; despite striking differences in resting sympathetic outflow, similar Mayer wave amplitudes were observed in older healthy males and young females (Fig. 3) [44].

Also, artificial augmentation of low-frequency pressure oscillations revealed a highly variable relationship between arterial pressure and cardiac interval fluctuations [45]. The increase in Mayer wave oscillations from graded forced oscillations support the implication of arterial baroreflex engagement. However, the strength of the cross-spectral coherence between low-frequency oscillations was highly unstable, within and among subjects, and across levels of forced oscillations. This indicates that heart period oscillations do not buffer pressure consistently through the baroreflex. Hence, even though baroreflex gain represents an important mechanism for cardiovascular oscillations at the Mayer wave frequency, other factors might also be involved.

### Measurement of heart rate variability

Changes in RRi defined as HRV indicate a normal response of the heart to multiple physiological and environmental stimuli such as breathing, physical exercise, mental stress, hemodynamic alterations, and metabolic changes [46, 47]. One way of understanding HRV is considering the variance with respect to the mean, i.e., a measure of the spread of the distribution. The fluctuations occur either in a random pattern (noise) or exhibit deterministic variations. As described above, changes in RRi reflect autonomic modulation and provide a sensitive and early indicator of health impairments. While high HRV is associated with efficient autonomic mechanisms in healthy individuals, low HRV is an indicator of abnormal and inadequate adaptations of the ANS and, in some cases, increased mortality and morbidity. Thus, HRV has been identified as a promising marker to study autonomic function and to diagnose pathological states, both in research and clinical setups. A range of indexes have been derived from fluctuations not just in heart period but also in blood pressure, sympathetic nerve activity, blood flow, “spontaneous” bareoreflex sensitivity, and cerebral "autoregulation". However, the significance and meaning of HRV is more complex than generally appreciated and careful examinations should be considered in measuring and interpreting it. Quantitative approaches in assessing HRV include linear methods, time domain, frequency domain, and nonlinear methods.

Electrocardiograms of variable durations have been used to assess HRV. In a continuous ECG record, the P wave representing the atrial contraction is the electrical signal that initiates a beat. The impulse is conducted through the atrioventricular node via the Purkinje fibers, leading to depolarization of the ventricles, which is represented by the Q, R, and S waves, forming the QRS complex in the ECG. Afterwards, the ventricular repolarization follows, represented by the T wave. During the R phase, most of the heart is activated resulting in the greatest wave shown by the ECG recording. Thus, most studies of cardiovascular variabilities use indexes of cardiac chronotropic response derived from the R wave in the QRS complex since it is easiest to detect. Heart rate variability indexes are obtained by analyzing the variation between beat-by-beat intervals measured between consecutive QRS complexes, conventionally named RRi. For assessing HRV, initial filtering methods are used to discard premature ectopic beats or artifacts, by detecting and correcting abnormal RRi.

### Linear methods

#### Time domain

In the time domain, indexes of HRV are expressed in unit time (ms). Statistical or geometrical methods (i.e., mean, standard deviation, and histogram-derived indexes) are used to assess fluctuations in the cardiac cycle from the inherently discrete and uneven time series. Statistical analysis of RRi in the time domain includes several indexes. The most routine measurements of HRV consider the mean of RRi for normal beats (mean RRi, in ms) and the standard deviation of all normal RRi (SDNN, in ms) recorded in a time interval. Other HRV indexes in the time domain include standard deviation of the means of RRi (SDANN, in ms), mean of the 5-min standard deviations of RRi (SDNNi, in ms), root-mean square of differences between adjacent normal RRi (rMSSD, in ms), and percentage of adjacent RRi with a difference of duration greater than 50 ms (pNN50).

The SDNN, SDANN, and SDNNi indexes are usually obtained from long-term recordings. About 30–40 % of the SDNN magnitude is attributed to day:night differences in RRi, and thus, long-term recordings of at least 18 h of data are required for accurate assessment [48, 49]. Correct calculation of these indexes require careful considerations, excluding ectopic beats, artifacts, and missed beats. These indexes can be used to assess autonomic nervous system activity; however, they cannot distinguish between changes in HRV due to increased sympathetic tone or withdrawal of vagal tone. The rMSSD and pNN50 indexes primarily quantify modulation of RRi driven by ventilation.

#### Frequency domain

Autoregressive models and the Fast Fourier Transform (FFT) are used to quantify cyclic fluctuations of RRi. Frequency domain analysis decomposes HRV into four fundamental oscillatory components seen as four peaks in the power spectra obtained using the FFT method. The high-frequency component (HF) represents a peak ranging between 0.15 and 0.40 Hz in the power spectra. As discussed above, the HF component usually reflects RSA, mostly due to vagal activity. Low frequency (LF) components range between 0.04 and 0.15 Hz (Mayer wave oscillations), occur over a period of 10 s, and are due to both sympathetic and parasympathetic control. The very low frequency (VLF) component ranges between 0.0033 and 0.04 Hz, or periods of 20 s to 5 min. The ultra-low frequency (ULF) component is associated with frequencies less than 0.003 Hz, with periods between 5 min and once during 24 h. The exact physiological mechanisms responsible for VLF and ULF are not yet established, but they may relate to the renin-angiotensin-aldeosterone system, thermoregulation, and/or peripheral vasomotor tone.

Further attempts to understand the complex mechanisms that underlie HRV include the use of the ratio of the high- and low-frequency oscillations (LF/HF) and/or the normalization of these two oscillations to total variability. The ratio of high- and low-frequency oscillations LF/HF is assumed to reflect the absolute and relative changes between the sympathetic and parasympathetic components. Normalization of the two oscillations is obtained by dividing the power of one of the components by the total power spectrum, and subtracting the VLF component. The conclusions derived from HRV when using normalized units and/or LF/HF ratio should be carefully reconsidered. The underlying assumption in using either normalized units and/or LF/HF ratio is that there is a sympathovagal balance that modulates the sinus node activity (i.e., increased activity in one system is accompanied by decreased activity in the other system). Support derives, in part, from the correlations between passive tilt angle in humans and normalized variability and the LF/HF ratio [50]. However, this reciprocal relationship between parasympathetic and sympathetic outflows does not apply to all conditions. For example, while only parasympathetic withdrawal occurs at low exercise intensities, both further parasympathetic withdrawal and sympathetic activation occur at moderate and higher exercise intensities in humans [51]. Moreover, normalizing variability data results in a change in total power (the denominator of the normalization) that can introduce artifactual changes and can lead to erroneous conclusion about the physiology of HRV. For example, high doses of atropine are parasympatholytic, markedly increasing heart rate and decreasing HRV. However, normalized units of variability indicate that significant oscillations still remain despite a nearly monotonic heart rate [52]. Nonetheless, when performed judiciously, spectral analysis can yield important insights, including delimiting frequency responses of the cardiovascular system, and potential cause relations among cardiovascular and respiratory variables.

### Non-linear methods

Non-linear phenomena are certainly involved in the genesis of HRV. The dynamic nature of HRV may not be properly appreciated by linear methods. As a result, theories of non-linear systems have been proposed as unique identifiers of the complex interactions of the numerous mechanisms that modulate variability in heart period. Hence, fractal mathematics and chaotic dynamics have been applied to HRV. The underlying assumption behind these techniques is that the individual behavior and dynamics observed represent the behavior of the overall system, independent of a characteristic time or length scale of operation. In healthy individuals, heart rate is neither constant nor strictly periodic. Instead, beat-to-beat HRV demonstrates an apparent chaotic pattern of fluctuations in healthy subjects. Time- and frequency-domain analysis of beat-to-beat heart rate oscillations show that these fluctuations could be chaotic in nature [53, 54]. Thus, it has been suggested that fractal measures of beat-to-beat time series of heart period could describe the integrated control of HRV independent of time or frequency scale [55, 56]. However, by definition, heart beats are discrete events, and thus, the resolution of the structure is limited and cannot be smaller than the shortest interval between consecutive beats. To correctly establish fractal behavior of a structure, the current literature suggests that large data series are necessary (at least 20 min) to allow reliable calculations of a single fractal scaling exponent [57, 58]. Even when some conditions are met to assume fractal behavior (such as a strong power-law scaling), a majority of heart period time series in healthy individuals do not conform to the assumed standard fractal model and thus cannot be considered fractal processes [59]. Moreover, the most relevant fluctuations in heart rate period occur around 6 s or faster (respiratory sinus arrhythmia, primarily related to vagal control) or around 10 s (Mayer wave oscillations, reflecting both sympathetic and parasympathetic control). Even when heart rate time series assume fractal behavior, given the restricted temporal range of heart rate fluctuations, it is important to assess the physiological meaning obtained from fractal analysis and their relevance to cardiac autonomic control. Thus, careful considerations should be made when using fractal estimates as the results might not reflect true changes in alterations within individuals, or they might produce physiologically meaningless and irreproducible values.

## Conclusions

Heart rate dynamically responds to internal and external perturbations on a beat-by-beat basis. The complexity of the heart rate time series has within it cardiovascular oscillations occurring at specific frequencies. However, the closed-loop nature of the control of these cardiovascular oscillations makes it difficult to define specific relations among autonomic variables. Both respiratory frequency oscillations (RSA) and slower oscillations at the Mayer wave frequency have been attributed to various feedforward and feedback mechanisms. Nonetheless, work in the area of cardiovascular variabilities has attempted to derive indexes for autonomic outflow from fluctuations in heart period and blood pressure. In particular, RSA has become a popular metric for autonomic control and is presumed by many investigators to have a one-to-one correspondence to cardiac vagal outflow. Even though vagal outflow is the dominant contributor to RSA, several observations presented in this review challenge the assumption that RSA is always a purely vagal mechanism or corresponds proportionately to vagal outflow. As with respiratory frequency oscillations, Mayer wave frequency variability has been presumed to represent a sympathetic effect on the heart. However, the exact relation between mean sympathetic outflow and Mayer wave amplitude is still unclear and it appears that cardiovascular oscillations at the Mayer wave frequency may be generated by mechanisms other than sympathetically mediated. Hence, the understanding of the integrated mechanisms that underlie these cardiovascular oscillations is still largely unknown and it is likely that they represent complex interactions of several effectors. Moreover, the measurement and quantification of these cardiovascular variabilities should be judiciously performed to yield meaningful physiological insights. Thus, given the complex nature of cardiovascular variabilities, careful consideration should be given when attempting to use them as quantitative measures of autonomic outflow or as clinical indexes of individual cardiovascular condition.

## Abbreviations

ACh: Acethylcholine
ANS: Autonomic nervous system
CVM: Cardio motor neurons
ECG: Electrocardiogram
HF: High frequency
HR: Heart rate
HRV: Heart rate variability
LF: Low frequency
RRi: R-R interval
RSA: Respiratory sinus arrhythmia
SA: Sinoatrial node
ULF: Ultra-low frequency
VLF: Very low frequency
